# Endophytic *Diaporthe* as Promising Leads for the Development of Biopesticides and Biofertilizers for a Sustainable Agriculture

**DOI:** 10.3390/microorganisms10122453

**Published:** 2022-12-12

**Authors:** Sandra Hilário, Micael F. M. Gonçalves

**Affiliations:** 1 Centre for Environmental and Marine Studies (CESAM), Department of Biology, University of Aveiro, Campus Universitário de Santiago, 3810-193 Aveiro, Portugal; 2 Division of Microbiology, Department of Pathology, Faculty of Medicine, University of Porto, 4200-319 Porto, Portugal

**Keywords:** antibacterial, antifungal, biofertilizers, endophytes, mycoherbicides, plant promoters

## Abstract

Plant pathogens are responsible for causing economic and production losses in several crops worldwide, thus reducing the quality and quantity of agricultural supplies. To reduce the usage of chemically synthesized pesticides, strategies and approaches using microorganisms are being used in plant disease management. Most of the studies concerning plant-growth promotion and biological agents to control plant diseases are mainly focused on bacteria. In addition, a great portion of registered and commercialized biopesticides are bacterial-based products. Despite fungal endophytes having been identified as promising candidates for their use in biological control, it is of the utmost importance to develop and improve the existing knowledge on this research field. The genus *Diaporthe*, encompasses plant pathogens, saprobes and endophytes that have been screened for secondary metabolite, mainly due to their production of polyketides and a variety of unique bioactive metabolites with agronomic importance. Some of these metabolites exhibit antifungal and antibacterial activity for controlling plant pathogens, and phytotoxic activity for the development of potential mycoherbicides. Moreover, species of *Diaporthe* are reported as promising agents in the development of biofertilizers. For this reason, in this review we summarize the potential of *Diaporthe* species to produce natural products with application in agriculture and describe the benefits of these fungi to promote their host plant’s growth.

## 1. Introduction

In 1807, Bénédict Prévost found that germination of spores from *Tilletia caries* was inhibited by metallic copper when placed in the soil, thus describing it as the first compound with fungicidal properties [1]. The first organic fungicide was synthesized in the early 20th century. After that, several fungicides such as 2-methoxyethyl silicate and 2-hydroxyphenyl mercury, effective against the fungal species *Fusarium* spp. and *Dreschlera* spp., started also to be commercialized [2]. Nevertheless, the excessive use of agrochemicals has contributed to the environmental pollution (e.g., long degradation period), undesirable effects on human health (e.g., carcinogenicity) and the development of pathogen resistance [3]. Therefore, alternative methods for the safe control of plant pathogens and weed managements such as the use of biocontrol microorganisms and the application of naturally sourced metabolites have received increasing attention in the past decade [4,5].

Microorganisms are known for their ability to synthesize secondary metabolites, which exhibit promising bioactivities for the development of agrochemicals. Many natural antifungal fungicides have been obtained from microbial resources [6]. For instance, kasugamycin isolated from *Streptomyces kasugaensis* is widely used to control leaf spot, fire blight, rice blast and bacterial diseases in several crops [7]. The polyoxins, produced by *Streptomyces cacaoi*, are effective for rice fungal diseases as well as for the gray mold disease of fruits (*Botrytis cinerea*) [8]. Moreover, the antifungal antibiotic validamycin produced by *Streptomyces hygroscopicus* var. *limoneus* is commonly used to control sheath blight of rice plants caused by *Rhizoctonia solani* [9]. It is also well-stablished that several fungal genera may confer herbicidal activities by producing competent phytotoxins, such as species of the genus *Colletotrichum* and *Xylaria* [10].

The application of endophytic fungi to promote a sustainable agriculture has also been of interest, due to their role as plant-growth promoters [11]. This role is based on recognized mechanisms, such as the increase in nutrient and water acquisition and the production of plant hormones, leading to an increase in resistance to biotic and abiotic stresses [12]. Recent research has also demonstrated that the use of bacteria and fungi as biological control agents is advantageous to control plant diseases, thus improving agricultural yields [13]. The application of fungal biological control agents has largely increased due to their high reproductive rate (sexually and asexually), and their being target specific [14].

The genus *Diaporthe* comprises plant pathogens and endophytes, and it is a source of secondary metabolites. These have been explored for their potential applications in health care (e.g., antioxidant and anti-inflammatory properties), pharmacology (e.g., clinical toxicology assessment) and biomedicine (e.g., development of drugs) [15,16]. However, there is still a lack of information on the phytotoxins produced by species of *Diaporthe*, which should be explored given their potential application in agriculture as promising candidates for the development of natural herbicides [15]. Moreover, endophytic *Diaporthe* species are also reported as producing antimicrobial compounds to control plant pathogens, and as promising agents in the development of biofertilizers to promote plant growth [17]. Therefore, the main goal of this review is to summarize the potential benefits of species of *Diaporthe* as biocontrol agents, and as promising sources for the development of antimicrobials and mycoherbicides to assist in a sustainable agriculture.

## 2. Material and Methods

### 2.1. Criteria Used for Considering Studies

This review was aimed to summarize and gather current knowledge from published scientific data concerning the importance of endophytic species of *Diaporthe* as biological control agents. Notwithstanding the recognition of *Diaporthe* as the most common genera of endophytic fungi, this research field still requires up-to-date review papers. Considering this, the literature review was organized and compiled to deepen the knowledge and identify the possibility of using endophytic *Diaporthe* as crucial elements for the development of biopesticides and biofertilizers to assist in a sustainable agriculture. The importance and suitability of multi-omics approaches on species of *Diaporthe* was also a key point discussed in this review. Multi-omics are important tools for unraveling functions and beneficial properties of endophytes and their metabolites. Moreover, such approaches are also crucial to unveil metabolic pathways towards plant growth and tolerance to environmental stresses. The assemblage of all published data covered in this review represents a step closer to sustainable and ecological agricultural production.

### 2.2. Search Strategy for Identification of Studies

The literature review was carried out mainly in Web of Science, Scopus and Google Scholar databases between 10 August 2022 to 15 October 2022. The main keywords used for this review were used alone or in combination as follows: endophyte, endophytic, *Diaporthe*, *Phomopsis*, omics, genomics, transcriptomics, metabolomics, proteomics, fungi, fungus, biocontrol, antagonism, microorganisms, biofertilizers, plant-microbe interactions, biopesticides, sustainable agriculture, agrochemicals, environment, phytotoxins and plant promoters. Only articles, reviews, book chapters and books written in the English language were included. Articles were also manually screened for additional references.

All scientific literature was taken into consideration with a special focus on publications from the last decade (2012–2022), which represents more than 77% of the references used (136 out of 176). However, to show evidence of the historical background of some topics covered in this review, some publications prior to the 2000s, dated between 1874 and 1999, were also selected which correspond to 7% of all references used (12 out of 176).

## 3. Fungal Endophytes and Their Benefits for Plants

**Figure 1:**
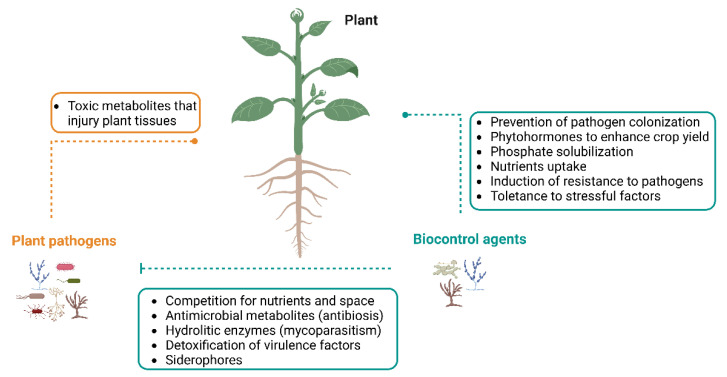
Overview of possible modes of action of endophytic fungi as biological control agents and plant-growth promoters. Beneficial microorganisms can exhibit direct antagonism against plant pathogens (inhibitor green line), as well as promote plant growth (dotted green line). Plant pathogens can also produce toxins to injure the plant (dot orange line). The figure was created with BioRender.com (accessed on 20 October 2022).

Although the term “endophyte” was originally introduced by de Bary in 1866, the most used definition of endophytes was proposed by Petrini in 1991 [18]. It refers to a group of organisms “inhabiting plant organs that at some time in their life can colonize internal plant tissues without causing apparent harm to the host” [19]. These endophytes are usually fungi or bacteria that are present in the phyllosphere, endosphere or rhizosphere. These microorganisms live in the tissues of plants without causing any symptoms of disease, leading to beneficial effects for the hosts (Figure 1) by:

(1) Facilitating the acquisition of limited nutrients (e.g., nitrogen) [3];
(2) Producing phytohormones (e.g., gibberellins and indole acetic acid) that enhance crop yield and quality [20,21,22];
(3) Providing plant tolerance to environmental stresses factors (e.g., salinity, drought, heavy metal presence) [3,20];
(4) Improving resistance to pathogens [3].

In this regard, some fungal species have been studied due to their ability to promote plant growth. For instance, *Fusarium equiseti* increased the herbage yield of *Trifolium subterraneum* (subclover) by facilitating nitrogen uptake, while *Sporormiella intermedia* increased the mineral uptake of calcium, copper and zinc in subclover, thus enhancing the nutritional value of forage [23]. Similarly, Baron et al. [24] used *Aspergillus sydowii* to inoculate maize plants (*Zea mays*). The authors stated that those plants inoculated with the fungus accumulated significantly higher amounts of phosphorous in their tissues. The endophyte *Colletotrichum tropicale* can also enhance the nitrogen uptake and change its distribution in cacao plants [25]. *Trichoderma asperellum* was also reported to significantly increase seed vigor and the yield of *Sorghum bicolor* roots [26].

Moreover, Khan et al. [27] detected gibberellin production by *Penicillium citrinum*. These authors have thus demonstrated that the *P. citrinum* improved the length of seedlings in the sandy plant *Atriplex gmelinii*, thus promoting its growth. Baron et al. [28] also demonstrated that the fungal species *Purpureocillium lilacinum*, *P. lavendulum* and *Metarhizium marquandii* are able to produce indole acetic acid and to solubilize phosphorous. The authors showed that these strains were able to promote the availability of phosphorous and nitrogen in soybean, bean and maize plants. In another study, Ismail et al. 2020 [29] reported that soybean plants inoculated with the endophyte *Aspergillus niger* showed tolerance to high temperatures. The inoculation with this fungal endophyte promoted and increased plant height, biomass and chlorophyll content, as well as to reduced lipid peroxidation during heat stress [29].

The intensive use of chemical fungicides to suppress the growth of plant pathogens over a long period have led to pesticide-related pollution, resistant microbial strains, chemical consumption through bioaccumulation, biodiversity losses and the elimination of natural/beneficial microorganisms [30]. Considering that the most current strategies contained in the United Nations 2030 Agenda (17 Sustainable Development Goals) aim at achieving sustainable development, the biopesticides application creates an equilibrium between economic productivity and environmental protection that is crucial to sustainable agriculture [30,31]. In this regard, the growing search for new biopesticides to replace synthetic chemicals is supported by its low toxicity properties, eco-friendliness, specificity, biodegradability, low post-harvest contamination and compatibility in integrated pest management [32].

The drawbacks of biopesticides usage are defined as the high cost of commercial products, standard method of preparations and dose determination of active substances [32]. Nevertheless, the application of antagonistic endophytic fungi as biocontrol agents, has drawn special attention for being a sustainable option for the management of some plant diseases, thus resulting in minimal impact on the environment [4,17,33]. The main interaction between endophytic fungi and pathogens is the limitation of mycelium growth by contact, or through the formation of inhibition zones in dual culture [34]. Such facts indicate that the endophytes that act as biocontrol agents harbor multiple mechanisms to control the pathogens (Figure 1) by:

(1) Competing for nutrients and space [35,36];
(2) Antibiosis-production of inhibitory metabolites or antibiotics [33,35,37];
(3) Induction of plant defense response against plant pathogens [35,38];
(4) Secretion of extracellular hydrolytic enzymes [38];
(5) Detoxification of virulence factors [38].

Since early times, man has attempted to increase and improve crop production and to control plant diseases by using antagonistic microorganisms [39]. For instance, Roberts, in 1874 [40], introduced the term antagonism in microbiology after showing the antagonistic action between the fungus *Penicillium glaucum* and a bacterial strain. Later in 1921, Hartley inoculated forest nursery soils with antagonistic fungi to control damping-off caused by *Pythium debaryanum* [41]. In 1941, Weindling [42] noted that species of *Trichoderma* produced an antifungal compound, the gliotoxin, that was toxic to plant pathogens including *Rhizoctonia solani* and *Sclerotinia americana*. This study conducted by Weindling [42] was the first to record the use of gliotoxin in plant disease control [43]. Since the discovery of penicillin by Alexander Fleming in 1928 with pharmaceutical application, the studies on the discovery of biological control agents against plant pathogens have been increasing, attempting to unveil secondary metabolites with promising applications in agriculture [14,44].

It is noteworthy that endophytic fungi produce large numbers of metabolites with different chemical structures from, including alkaloids, terpenoids, benzopyranones or quinones [45]. These compounds are crucial for agricultural application once they exhibit promising bioactivities such as antifungal, antibacterial, herbicidal and other agricultural activities [3,16]. For instance, the fungal genus *Xylaria* associated with the *Azadirachta indica* plant produces antifungal compounds with activities against *Aspergillus niger* and *Fusarium avenaceum* [46,47]. Sangeetha et al. [48] demonstrated that species of *Trichoderma* may produce antifungal compounds due to their biocontrol potential against *Colletotrichum musae*, *Fusarium verticillioides* and *Lasiodiplodia theobromae* (causing postharvest crown rot of banana). Griseofulvin, a secondary metabolite initially isolated from the fungus *Penicillium griseofulvum*, has drawn special attention due to many reports of antifungal activities against plant pathogenic fungi such as *Cytospora* sp., *Cladosporium gloeosporioides*, *Botrytis cinerea*, *Alternaria solani* and *Fusarium solani* [49]. Therefore, endophytic fungi are promising leads for the discovery of novel secondary metabolites with potential for agricultural applications as biocontrol agents, biostimulants, biofertilizers and bioherbicides [3,4,13,50].

## 4. Species of *Diaporthe* as Benefit Microorganisms to Agriculture

### 4.1. Production of Antimicrobial Compounds

Species of the genus *Diaporthe* can switch between lifestyles, meaning that the same species can be found on the same or other hosts as phytopathogens or as endophytes in asymptomatic tissues [51,52]. For example, *D. eres* is a pathogen that is present on a wide range of hosts, including economically important fruit trees (e.g., apple, blueberry, hazelnut) [53,54,55] and ornamental plants (e.g., *Allium giganteum*, *Magnolia soulangeana*) [56], and as an endophyte on *Prunus domestica* [17]. Moreover, *D. limonicola* can be found on *Citrus grandis* leaves as an endophyte [57], or as a pathogen causing dieback on lemon trees in Europe [58].

Despite its known record as a plant pathogen, *Diaporthe* is recognized as one of the most frequently isolated genera occurring as endophytes in the stems and leaves of several hosts in tropical and temperate ecosystems [55,56,59]. Due to the high number of species of *Diaporthe* as endophytes, and given their potential as producers of secondary metabolites, these species have been widely investigated for the production of valuable compounds with different bioactivities [17]. A recent review by Xu et al. [16] summarized a total of 335 bioactive secondary metabolites isolated from species of *Diaporthe* and Phomopsis-like species. These metabolites were classified into polyketides, terpenoids, steroids, macrolides, ten-membered lactones, alkaloids, flavonoids and fatty acids. Polyketides are the main chemical population (64%), and their bioactivities involve antitumor (e.g., clavaric acid), antioxidant (e.g., pyranonigrin E; diportharine A; phochrodine D) [60,61], cytotoxic (e.g., diaporthelactone, phomopsidone A, phomaspyrone A-E) [62,63], anti-bacterial (e.g., phomosine A, 3-Hydroxypropionic acid) [64,65], anti-fungal (e.g., (+)-2,2′-Epicytoskyrin A, phomopsolide A-C) [66,67,68], antimalarial (e.g., epoxycytochalasin H) [69] and anti-inflammatory activities (e.g., Biatriosporin N) [70]. Considering the several compounds produced by species of the genus *Diaporthe*, Xu et al. [16] have stated that this genus is a promising source for the discovery of small molecules for drug candidates.

In agriculture, several studies have reported that species of the genus *Diaporthe* (including Phomopsis-like species) also exhibit antibacterial and antifungal activity against plant pathogenic microorganisms (Table 1). For instance, Abramczyk et al. [17] showed that *D. eres* from *Prunus dulcis* exhibited antifungal activity against *Trichothecium roseum*, *F. avenaceum* and *A. alternata*. Moreover, Endophytic species of *Diaporthe*, isolated from *Pachystachys lutea*, were effective against *F. oxysporum* and *Colletotrichum* sp. [34]. The antifungal activity of *D. citri*, isolated from *Mikania glomerata*, was also verified against *F. solani* and *Didymella bryoniae* [71]. Verma et al. [72] also demonstrated that under dual culture techniques, *D. melonis* and *D. longicolla* showed antagonism to *Corynespora cassiicola* and *F. solani* with an inhibition halo percentage ranging from 52–64% against *C. cassiicola* and 28–52% against *F. solani*.

Moreover, two derivatives of the phytotoxin alternariol, alternariol 4,10-dimethyl ether and alternariol methyl ether, were isolated from *Diaporthe phragmitis* (syn. *D. eres*), a kiwi endophytic fungus [73]. The authors showed that this endophytic fungus exhibited antibacterial activity against *Pseudomonas syringae* pv. *actinidiae* which causes kiwi cankers. Carvalho et al. [74] have also shown that seven strains of *D. miriciae* and two strains of a *Diaporthe* sp. produced cytochalasins H and J. After a bioassay-directed fractionation to isolate cytochalasins, the authors evaluated the compounds for activities against the fungal plant pathogens *Colletotrichum fragariae*, *Botrytis cinerea*, *F. oxysporum, Phomopsis obscurans* (syn. *Paraphomopsis obscurans*) and *Phomopsis viticola* (syn. *D. rudis*). The cytochalasins H and J exhibited effective activities against *Paraphomopsis obscurans*, associated with strawberry leaf blight and *D. rudis*, a grapevine pathogen [74]. Therefore, these studies suggest that *Diaporthe* fungal endophytes could be used as biocontrol agents and offer insights for the screening and isolation of antimicrobial compounds for the further development of new agrochemicals.

**Table 1:** Antimicrobial activity of *Diaporthe* against fungal and bacterial plant pathogens.

Host Plant	Endophyte	Antimicrobial Activity	References
*Aconitum* *carmichaelii*	*Diaporthe* sp.	Antifungal activity against the rice blast fungus *Magnaporthe oryzae*	[75]
*Aconitum carmichaeli*	*D. amygdali*	Antifungal activity against plant pathogenic fungi: *Fusarium graminearum*, *Verticillium albo-atrum* and *Microdochium nivale*	[76]
*Actinidia chinensis*	*D. phragmatis* (syn. *D. eres*)	Inhibitory activity against *Pseudomonas syringae* pv. *actinidiae*, the causal agent of kiwi canker disease	[73]
*Balanophora polyandra*	*D. foeniculina*	Antibacterial potential against plant pathogenic bacteria: *Ralstonia solanacearum*, *Pseudomonas lachrymans* and *Xanthomonas vesicatoria*	[77]
*Cistus salvifolius*	*Diaporthe* sp.	Antifungal activity against pathogens of agricultural importance: *Phytophthora infestans*, *Botrytis cinerea* and *Septoria tritici*	[78]
*Copaifera* *pubiflora* and *Melocactus ernestii*	*D. miriciae*	Antifungal activity against the grapevine pathogen *D. rudis*	[74]
*Endodesmia calophylloides*	*Diaporthe* sp.	Inhibition of zoospores of grapevine pathogen *Plasmopara viticola*	[79]
*Espeletia* sp.	*D. phaseolorum*	Antifungal activity against the plant pathogen *Phytophthora infestans*	[80]
*Gossypium hirsutum* and *G. arboreum*	*D. longicolla* and *D. melonis*	Antifungal activity against *P. citricarpa*	[72]
*Gossypium hirsutum*	Phomopsis-like species	Antifungal activity against *Sclerotinia sclerotiorum*, *F. oxysporum*, *B.* *cinerea*, *Bipolaris sorokiniana*, *Gaeumannomyces graminis* var. *tritici*, and *Rhizoctonia cerealis*	[81]
*Maytenus ilicifolia*	*D. endophytica*	Antifungal activity against the citrus pathogen *Phyllosticta citricarpa*	[82,83]
*Mikania glomerata*	*D. citri*	Antifungal activity against *F. solani* and *Didymella bryoniae*	[71]
*Pachystachys lutea*	*Diaporthe* sp.	Antifungal activity against the pathogenic fungi *F. oxysporum* and *Colletotrichum* sp.	[34]
*Prunus domestica*	*D. eres*	Antifungal activity against plant pathogenic fungi: *Trichothecium roseum*, *F. avenaceum* and *Alternaria alternata*	[17]
*Rhizophora mucronata*	*Diaporthe* sp.	Antifungal activity against pathogens of agricultural importance: *Verticillium dahlia*, *Botrytis cinerea* and *Sclerotinia sclerotiorum*	[84]
*Schinus terebinthifolius*	*D. terebinthifolii*	Antifungal activity against the citrus pathogen *Phyllosticta citricarpa*	[82,83]
*Solanum lycopersicum*	*D. phaseolorum*	Inhibitory activity against bacterial spot of tomato (*Xanthomonas vesicatoria*)	[85]
*Vochysia divergens* and *Stryphnodendron adstringens*	*Diaporthe* cf. *heveae*	Antifungal activity against *P. citricarpa* and *Colletotrichum abscissum*	[86]

### 4.2. Phytotoxins as Potential Mycoherbicides

Weeds hamper the growth of several crops once they compete with the plants for water and nutrients resulting in enormous production losses [87]. Moreover, weeds can act as a host for insects and pathogens (fungi and bacteria), which can cause serious damage to crop plants [88]. Therefore, weeds management is a crucial agricultural practice to avoid significant yield losses [89]. However, chemical herbicides can have negative side effects, such as surface and ground water contamination, leaving herbicide residues in the food chain and, decreasing the soil microbial communities and earthworm populations, thus suppressing nutrient availability and soil biodiversity. [87,90]. Therefore, the use of herbicides should be minimal and effective to reduce their impact on human health and the environment. Mitigation of herbicides should be considered an important achievement for a sustainable agriculture. [90]. Such facts drive a growing search for new herbicides with low toxicity profiles, which is a step closer for human safety and environmental health [87,91]. One important aspect to be highlighted is that bioherbicides do not need to cause the death of weeds to increase crop productivity; they can suppress weed populations, which is a strategy with low risk when compared with the application of chemical herbicides [92].

Among the several microorganisms producing phytotoxins, some of them exhibit a potential for the production of molecules with herbicidal activity. Accumulating evidence on molecular biology and natural products demonstrates that many fungal species are a promising source of natural phytotoxins (e.g., *Alternaria*, *Colletotrichum*, *Chondrostereum, Lasiodiplodia, Sclerotinia, Xylaria*) [10,93]. For example, phytotoxic compounds isolated from the endophytic *Xylaria feejeensis* exhibited a toxic effect on the photosynthesis machinery of spinach chloroplasts [94].

Species of the genus *Diaporthe* (including Phomopsis-like species) are also producers of phytotoxins, showing interesting results (Table 2) [92,95,96,97,98,99]. For instance, Cimmino et al. [100] tested the fungal phytotoxin phomentrioloxin produced by *D. gulyae* and verified a suppression in the growth of the annual weed *Carthamus lanatus*. Brun et al. [92] have also demonstrated that biomolecules from *D. schini* caused yellowing lesions and a decrease in the height of the grass species *Echinochloa crusgalli* and *Lolium multiflorum*. Kongiidiazadione, isolated from *D. kongii*, showed a phytotoxic activity on the leaves of tomato plants but caused clear and significant necrosis on *H. annuus* [101]. Additionally, an endophytic *Phomopsis* sp. (*Diaporthe*) was reported by Yang et al. [102] as the producer of phytotoxins such as cytochalasins (H, N, and epoxycytochalasin H) and a nonenolide compound ((6S,7R,9R)6,7-Dihydroxy-9-propylnon-4-eno-9-lactone). The authors demonstrated that the above-mentioned compounds showed phytotoxic effects on *Medicago sativa*, *Trifolium hybridum* and *Buchloe dactyloides* by decreasing the germination and radicle growth [102]. Such examples are evidence that species of *Diaporthe* are a source of phytotoxic compounds. Therefore, it is crucial to analyze, screen and isolate herbicidal compounds from these species to be applied in the development of new mycoherbicides.

### 4.3. Plant-Growth Promoters

Chemical fertilizers are used to boost agricultural productivity but can cause negative environmental impacts [115]. In addition, environmental stresses and climate changes scenarios (e.g., drought, high soil salinity) are major limitations to plant growth and yield, which can cause production losses [116,117]. For this reason, to counteract potential losses, endophytic fungi are an alternative to increase agricultural productivity. Endophytic microorganisms, which inhabit plant tissues, are capable of increasing agricultural productivity by increasing access to nutrients (e.g., nitrogen, phosphorus, potassium, zinc, iron), production of phytohormones or by increasing the water acquisition rates [115,118]. Among endophytic fungi used as biofertilizers, *Epichloë bromicola,* for instance, increases seed germination and growth capacity of wild barley (*Hordeum brevisubulatum*) when exposed to salinity stress [6]. Additionally, *Piriformospora indica*, an endophyte with high economic importance, are able to enhance nutrient uptake and to modulate the phytohormones involved in the growth and development of *Hordeum vulgare* [119].

Species of the genus *Diaporthe* are also well documented promising agents in the development of biofertilizers due to their plant-growth properties (Table 3). For example, the generalist fungus *Phomopsis liquidambaris* (syn. *D. liquidambaris*) is also able to establish symbiosis with rice and peanut plants by colonizing their roots and conferring tolerance to abiotic stress, as well as promoting plant host growth [120,121]. This species is also reported to induce rice resistance to *Fusarium graminearum*, the causal agent of rice spikelet rot disease [122]. Recently, Aldana et al. [123] reported that root and shoot biomass of the hybrid *Triticum durum* × *Hordeum chilense* increased by up to 30% after inoculation with a strain of *Diaporthe* sp. The authors have also shown increased concentrations of calcium, magnesium, sulphur, iron and boron in inoculated plants. Additionally, the inoculation of *Triticum durum* × *Hordeum chilense* with *Diaporthe* sp. strain EB4 under salinity conditions, heightened proline, gibberellins, and indole 3-acetic acid and increased nutrient uptake in roots, thus resulting in an enhanced growth [124]. Moreover, Ważny et al. [125] stated that the inoculation of *Noccaea goesingensis* with *Diaporthe eres* improved the biomass of the plant and increased nickel accumulation. The authors have thus proposed *D. eres* as a nickel uptake stimulating microorganism, which might be potentially used as a biofertilizer and in the bioremediation of metal contaminated soils.

## 5. Omics to Explore the Secondary Metabolism of *Diaporthe*

### 5.1. Genomics

**Figure 2:**
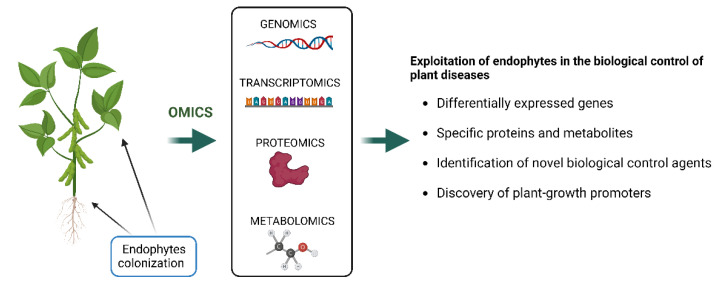
Schematic representation of omics approaches’ suitability for the exploitation of fungal endophytes for their application as biocontrol agents and plant-growth promoters. The figure was created with BioRender.com (accessed on 20 October 2022).

The application of omics approaches including genomics, transcriptomics, proteomics and metabolomics, opens up a new opportunity for the discovery of novel genes and their functions, novel pathways and metabolic network [134] (Figure 2).

Next-generation sequencing has been widely used to identify and characterize genes involved in plant–endophyte interactions [135]. Genome analysis has revealed genes responsible for nitrogen fixation, nutrition acquisition, and hormones biosynthesis as well as has elucidated the adaptative genomic signatures for bioactive secondary metabolites [136]. Recent genome mining has also offered in-depth information to search for natural products from fungi, and their biosynthetic gene clusters (BGCs) involved in different biosynthetic pathways [137,138]. Since 2011, researchers have used the “antibiotics and secondary metabolite analysis shell–antiSMASH” for their microbial genome mining tasks [139]. In fungi, less than 3% of the biosynthetic space of fungal genomes has been linked to the production of secondary metabolites, which are encoded by BGCs. The most common BGCs include non-ribosomal peptide (NRPSs), polyketide (PKSs) and terpene synthases [140]. However, the number of genomes available from the genus *Diaporthe* hampers the identification of BGCs and thus the discovery of bioactive compounds from these species. A search at NCBI (National Center for Biotechnology Information) (https://www.ncbi.nlm.nih.gov/, accessed on 19 June 2022) and JGI Genome Portal (Joint Genome Institute, Berkeley, CA, USA) (https://genome.jgi.doe.gov/portal/, accessed on 19 June 2022), unveiled 20 species of *Diaporthe* that have completed or are undergoing genome projects.

In this review, species with genomes available at the NCBI database were screened for BGCs (Table 4) and we identified some important compounds with 100% similarity with known BGCs with potential agricultural application The BGCs were predicted using the web-based application antiSMASH v.5.0, using the strictness ‘relaxed’ option [140].

Alternariol is classified as a phytotoxic and antifungal compound produced by species of the genus *Alternaria*, an important contaminant in cereals [141]. Nevertheless, its complete BGC has been detected in the genomes of *D. amygdali*, *D. destruens*, *D. capsici*, *D. citri*, *D. citrichinensis*, *D. eres* (syn. *D. phragmitis*) and *D. eres* (syn. *D. vaccinii*). This suggests the ability of these species to efficiently produce alternariol, and consequently their status as promising sources for the development of biopesticides. Moreover, (-)-Mellein is a phenolic compound initially isolated from *Aspergillus melleus* that showed antimicrobial activity [142]. Therefore, it is suggested that this compound could be screened and isolated from species of *Diaporthe* and produced to inhibit the growth of competitors and thus take on an agricultural application.

Fusicoccin A is a remarkable phytotoxin produced by the fungal species *Phomopsis amygdali* (syn. *D. amygdali*) [109,130]. However, it has been shown that it can be more effective than the growth-promoting hormone auxin [131]. Balio et al. [143] were prompted to investigate the effect of Fusicoccin in plants and carried out a pilot-scale study for the production of cultures of the fungus [143]. The quantities of the toxin available unveiled the physiological effects enhanced by Fusicoccin A such as an increase in tissue growth, nutrient uptake and the breaking seed coat dormancy [143,144]. Considering that the BGC that encode for this toxin was detected in the genome of *D. amygdali* with 100% similarity (Table 4), we can suggest that besides its reported phytotoxic activity, Fusicoccin can be used as a plant-growth promoter [15,130].

Asperlactone belongs the methylsalicylic acid (MSA) type polyketide group and has strong antibacterial and antifungal activities [145,146]. It has been reported that asperlactone also presented ovicidal activities [147] against *Nezara viridula*, a threatening pest for agriculture [148], and *Tribolium castaneum*, a significant global pest of stored food [149]. Considering that *D. longicolla* contains the complete BGC that encode for asperlactone, it is suggested that this species is able to produce this compound and should thus be regarded as a promising source for the development of novel bioinsecticides.

However, the presence of the complete BGC involved in the biosynthesis of one particular compound does not necessarily imply the production of that compound. Mainly because sometimes BGCs may remain silent under laboratory conditions [150]. Therefore, to bridge this flaw, altering cultivation parameters or adding chemical elicitors to activate silent BGCs can elucidate the hidden reservoir of complex chemical diversity. Such an outcome is readily achieved by the one strain many compounds (OSMAC) approach [151,152]. For instance, Zhang et al. [150] used the OSMAC approach on *Aspergillus fumigatus* LN-4 and *Xylaria* sp. XC-1 for the isolation of anticancer compounds and phytotoxins, respectively, by altering the cultivation parameters.

**Table 4:** Some secondary compounds produced by species of *Diaporthe* which are 100% identical to known BGCs.

Compound	Compound Nature	Biological Function	Species	References
ACR-Toxin I	Polyketide	Phytotoxin produced by the plant pathogenic fungus *A. alternata*, causing lemon leaf spot disease.	*D. ampelina* *D. helianthi*	[153,154]
ACT-Toxin II	Polyketide	Toxin causing brown spot disease on tangerine, produced by *A. alternata*	*D. eres* *D. capsici* *D. citrisiana* *D. vaccinii* (syn. *D. eres*)	[155]
Alternariol	Polyketide	Metabolite produced by *Alternaria spp.* that exhibits both phytotoxic and antifungal activity (e.g., zoosporicidal potential)	*D. amygdali* *D. destruens* *D. capsici* *D. citri* *D. citrichinensis* *D. phragmitis* (syn. *D. eres*) *D. vaccinii* (syn. *D. eres*)	[141]
Asperlactone	Polyketide	Methylsalicylic acid produced by *Aspergillus westerdijkiae,* antibacterial, antifungal and insecticidal activities	*D. longicolla*	[145,146]
Enniatin	Non-ribosomal peptide	Toxin produced by *Fusarium* spp., as a contaminant in cereals. It is also known as antibacterial, antifungal, and herbicidal.	*D. citrichinensis*	[156,157]
Fusarin	Polyketide	Mycotoxin produced mainly by fungi of the genus *Fusarium*, which can infect agriculturally important crops.	*D. amygdali* *D. aspalathi* *D. helianthi*	[158,159]
Fusicoccin	Terpene	Phytotoxin produced by *D. amygdali*, with plant-growth promoting potential	*D. amygdali*	[131]
(-)-Mellein	Phenolic compound	Metabolite firstly isolated from *Alternaria melleus*, with phytotoxic and antimicrobial activity	*D. capsici* *D. citri* *D. citrichinensis* *D. destruens* *D. phragmitis* (syn. *D. eres*) *D. vaccinii* (syn. *D. eres*) *D. longicolla*	[142]

Besides the presence of BGCs that have 100% homology with known compounds, it also detected some incomplete BGCs. For instance, on *D. amygdali* CAA958 and *D. eres* (syn. *D. vaccinii*) CBS 160.32 genomes, the BGC encoding for betaenones (phytotoxic polyketides) was detected. By analyzing the BGCs, we found that both species contain four genes encoding for enzymes involved in betaenone synthesis but were lacking a dehydrogenase and a FAD-dependent oxidase [160].

The BGC encoding for chaetoglobosin X, a hybrid polyketide with antifungal activity, was detected on the genome of *D. citrichinensis* ZJUD34. From the 11 genes involved in the biosynthesis of this compound, only 7 genes were detected such as *PN3-12* (C6 zinc finger protein), *PN3-13* (hybrid NRPS/PKS), *PN3-14* (enoyl reductase), *PN3-15* (hypothetical protein), *PN3-16* (FAD-linked oxidoreductase), *PN3-17* (P450 monooxygenase) and *PN3-19* (P450 oxygenase). Given that four of these genes present in *D. citrichinensis* are identified as being involved in the biosynthesis of chaetoglobosins [161], it is suggested that this species may have the potential to produce a related compound.

Nevertheless, on both betaenone and chaetoglobosin BGCs, the absent genes needed to complement the incomplete cluster may be silent or truncated, or even located on different fragmented contigs. The absence of those necessary genes for the core cluster suggests that many BGCs may not be functional and thus require additional experimental validation [162].

### 5.2. Transcriptomics

While genomics provides the whole genomic sequencing information, transcriptomics identifies the genes with changed expressions under a particular condition. It also involves the comparative analysis of the transcriptomes of groups and aids to understand the response of microbial communities upon changing environments [135,163].

The regulation of transcripts can be achieved by transcriptomic analysis. For example, mRNA sequencing is a valuable approach to understand differences in plants’ response in the presence and absence of endophytes [135]. In this regard, Ważny et al. [125] compared the transcriptomes of the hyperaccumulating plants *Noccaea caerulescens* and *N. goesingensis,* inoculated with *Phomopsis columnaris* (syn. *D. eres*), with uninoculated controls. The authors showed that the presence of the fungus enhanced uptake and accumulation of nickel, as well as that several genes involved in plant stress protection and metal uptake were upregulated.

Moreover, to understand the mechanisms of plant colonization by *Phomopsis liquidambaris* (syn. *D. liquidambaris*) under low nitrogen conditions, Zhou et al. [122] performed a transcriptomic analysis by the RNA-Seq technique. The authors compared the transcriptome profiles of inoculated and non-inoculated rice plants, and observed that gibberellin and auxin related genes, as well as genes encoding plant defense-related endopeptidase inhibitors, were upregulated on inoculated plants. Such results using transcriptomics might be useful to provide a better understanding regarding the molecular mechanisms of plant-endophyte interaction, as well as a deeper knowledge of fungal endophytes that promote plant growth under different conditions.

### 5.3. Proteomics

Nowadays, mass spectrometry (MS)-based proteomics is an effective tool to map the proteome of fungal endophytes, providing a solid basis for understanding the mechanisms involved in plant–microbe interactions (e.g., biological pathways and posttranslational modifications) [164]. The protein content of uninoculated and endophyte-inoculated plants can be predicted and assessed to investigate which specific proteins are involved in the relationship between these two groups. For instance, Yuan et al. [165] unveiled the transcriptome and proteome of *Atractylodes lancea* inoculated with and without the endophytic fungus *Gilmaniella* sp. AL12. In the study, the authors observed an upregulation of proteins involved in carbon fixation, carbohydrate metabolism and energy metabolism, thus suggesting that *Gilmaniella* sp. may improve the biomass of the herbal medicine *A. lancea* [165].

Nevertheless, as far as we know, no proteomic studies have been conducted in the genus *Diaporthe*. As the proteome profiling allows us to examine the structures, functions and interactions of dynamic proteins in an organism under a specific condition [135,166], in-depth studies should be undertaken to reveal the mechanism of the interactions between species of endophytic *Diaporthe* and their hosts.

### 5.4. Metabolomics

The discovery of new secondary metabolites that are involved in cellular functions and microbial networking can assist in the development of alternative antibiotics through microbial metabolite profiling [167]. The analysis of the diverse metabolites has been performed using mainly the following techniques: CE-MS (capillary electrophoresis), LC-MS and GC-MS (liquid and gas chromatography with mass spectrometry) and NMR (nuclear magnetic resonance spectroscopy) [168].

Several studies demonstrate that the genus *Diaporthe* have the potential to produce a wide range of metabolites with several biological activities with applications mainly in pharmaceuticals and biomedicine [169]. For instance, Kemkuignou et al. [169] used metabolomics approaches, mainly the High-Resolution ElectroSpray Ionization Mass Spectrometry (HR-ESIMS) to screen for the presence of metabolites from the species *D. breyniae*. The authors extracted and isolated the polyketides fusaristatin G and H, and the cytochalasan phomopchalasin N. Kemkuignou et al. [169] have also demonstrated the antimicrobial activity of these compounds against the fungus *Mucor hiemalis*, the bacteria *Staphylococcus aureus* and the yeast *Schizosaccharomyces pombe*. Moreover, through semi-preparative high-performance liquid chromatography (HPLC), Yedukondalu et al. [170] extracted and separated the compounds diapolic acid A-B and xylarolide from *D. terebinthifolii*. The authors demonstrated that diapolic A-B exhibited strong antibacterial activity against the human pathogen *Yersinia enterocolitica*, while xylarolide was effective against *Candida albicans* and showed potent cytotoxicity against the breast cancer cell line. Moreover, through the NMR approach, Mandavid et al. [171] isolated the polyketide mycoepoxydiene from the fungus *D. pseudomangiferae*. This metabolite was shown to exhibit cytotoxic activity against some human cancer cell lines (e.g., uterine cervical carcinoma, breast cancer and lung fibroblast) [171].

Although metabolomics is useful in showing the relationship among plants and growth promoters [172], this approach has been less frequently employed in determining functional traits in endophytic microbes [173]. For instance, Cao et al. [174] used mass spectrometry to unveil the metabolic profiles of the endophytic fungus *Neotyphodium lolii* and its host ryegrass (*Lolium perenne*). The authors verified changes in the metabolome of inoculated plants and reported the presence of key compounds such as peramine and perloline (alkaloids that increase the capacity of plants to resist the environmental stresses).

Similarly, our understanding of the functional secondary metabolites of species of *Diaporthe* on microbial interactions in plants are still scarce. Lacerda et al. [127] applied the NMR technique to report the metabolomic changes in *Combretum lanceolatum* plants inoculated with the endophytic fungus *Diaporthe phaseolorum*. The authors demonstrated that this fungus affected the metabolic pathways of the plant aerial parts, improving the biosynthesis of primary metabolites involved in plant self-defense such as threonine, malic acid and N-acetyl-mannosamine [127]. Therefore, to better understand the mechanisms of plant–*Diaporthe* interactions, studies using integrated analysis of genomics, transcriptomics, proteomics and metabolomics could be crucial to analyze key genes, specific proteins and metabolites that are differentially expressed. The exploitation of endophytes is surely important for the identification of biological control agents, as well as of plant-growth promoters, to assist in a sustainable agriculture (Figure 2).

## 6. Conclusions and Future Prospects

Regardless of the recognized benefits of endophytic fungi on plants and their potential in both biocontrol and biofertilization, they have been rarely studied regarding their application in agriculture. Nevertheless, due to the actual climate change scenarios (e.g., drought and high levels of soil salinity), it is crucial to understand the impacts of these environmental stresses on agriculture, as well as to unravel adaptation patterns of the endophytic community. Moreover, an effective utilization of endophytic fungi aids in promoting a sustainable agriculture for a safe environment and a positive impact on human health.

In this review, our results demonstrate that phytotoxic compounds, antibacterial and antifungal metabolites from species of the genus *Diaporthe* are promising leads for the development of new biopesticides. This study also suggests that species of this genus could be used as biofertilizers, given their ability as plant-growth promoters and stress tolerance-enhancers. However, a deeper understanding is needed not only to unveil which adaptation patterns of *Diaporthe* are triggered for their adaptation to changing climatic conditions, but also which adapted community might be applied as tolerance-enhancer treatment. This can be achieved through omics approaches that have revealed an enormous potential to unravel the functions of endophytes and their metabolites in plant disease control, as well as metabolic pathways towards plant growth and tolerance to environmental stresses. Moreover, multi-omics offer a valuable framework that allows a detailed analysis of the biological mechanisms of endophytic *Diaporthe* to design strategies highlighting their beneficial properties as control agents and plant-growth promoters. Therefore, a detailed understanding of species of *Diaporthe*, their mechanisms of action and bioactive metabolites, will likely provide a strong basis for developing:

(1) Reliable tools to enhance plant health and growth;
(2) Novel strategies for mitigating the impacts of climate changes;
(3) An ecological and sustainable agriculture.

In recent years, accumulating evidence has provided important advances regarding biological control agents for the development of commercialized bacterial and fungal-based biopesticides to control plant diseases. However, the implementation of large-scale studies to expand the knowledge on the usage of biopesticides is still hampered by the high cost of commercial products, the standard methods of preparations, the dose determination of active substances and the susceptibility of biopesticides to environmental conditions. In this regard, and taking into consideration the possibility of using endophytic *Diaporthe* as promising leads for the development of biopesticides and biofertilizers, some strategies should be adopted to improve the performance of these endophytic fungi. For instance, the development of specific delivery systems such as biopriming, encapsulation or foliar spraying should be favored to support the success of biocontrol and biofertilization programs. Moreover, the development of effective microbial consortium composed of endophytic fungi such as the species of *Diaporthe* could also be a promising strategy, not only to ensure the microbial diversity in the soil, but also in the phylosphere; phylosphere colonization is of paramount importance to ensure crop development and plant health management, regulating plant physiology under climate change scenarios.

## Figures and Tables

**Table 2:** Overview of some secondary metabolites isolated from species of *Diaporthe* and *Phomopsis*-like species with phytotoxic activity.

Compound	Strain	Host	References
3,4-Dihydro-8-hydroxy-3,5-dimethylisocoumarin	*D. eres*	*Hedera helix*	[103]
(6S,7R,9R)6,7-Dihydroxy-9-propylnon-4-eno-9-lactone	*Phomopsis* sp. (syn. *Diaporthe* sp.)	*Achyranthes bidentata*	[100,102]
4,6-dihydroxymellein	*P. helianthi* (syn. *D. helianthi)*	*Helianthus annus*	[104]
4-Hydroxybenzaldehyde	*D. eres*	*Vitis vinifera*	[105]
4-Hydroxybenzoic acid	*D. eres*	*Vitis vinifera*	[105]
8-hydroxy-3,7-dimethylisochroman-1-one	*D. eres*	*Hedera helix*	[99]
5-(hydroxymethyl) mellein	*Phomopsis* sp. (syn. *Diaporthe* sp.)	*Musa acuminata* *Cistus monspeliensis*	[106,107]
2-(4-hydroxyphenyl)-ethanol	*D. eres*	*Hedera helix*	[99,103]
5-methylmellein	*Phomopsis* sp. (syn. *Diaporthe* sp.)	*Musa acuminata* *Cistus monspeliensis*	[106,107]
3-Nitropropionic acid	*D. gulyae*	*Carthamus lanatus*	[95]
alternariol 4,10-dimethyl	*P. phragmitis* (syn. *D. eres*)	*Actinidia chinensis*	[73]
Altersolanol A	*P. foeniculi* (syn. *D. angelicae*)	Foeniculum vulgare	[108]
Altersolanol J	*P. foeniculi* (syn. *D. angelicae*)	*Foeniculum vulgare*	[108]
Convolvulanic acid A	*P. convolvulus* (syn. *D. convolvuli*)	*Convolvulus arvensis*	[109]
Convolvulanic acid B	*P. convolvulus* (syn. *D. convolvuli*)	*Convolvulus arvensis*	[109]
Convolvulol	*P. convolvulus* (syn. *D. convolvuli*)	*Convolvulus arvensis*	[109]
*p*-Cresol	*D. eres*	*Vitis vinifera*	[105]
Cytochalasin H, N	*Phomopsis* sp. (syn. *Diaporthe* sp.) *D. miriciae*	*Achyranthes bidentata*, *Copaifera* *pubiflora* and *Melocactus ernestii*	[74,102]
dideacetylfusicoccin	*P. amygdali* (syn. *D. amygdali*)	-	[110]
Epoxycytochalasin H	*Phomopsis* sp. (syn. *Diaporthe* sp.)	*Achyranthes bidentata*	[102]
etheralternariol methyl ether	*P. phragmitis* (syn. *D. eres*)	*Actinidia chinensis*	[73]
Foeniculoxin	*P. foeniculi* (syn. *D. angelicae*)	*Foeniculum vulgare*	[111]
Fusicoccin	*P. amygdali* (syn. *D. amygdali)*	*Prunus dulcis* *Prunus persica*	[112]
Gulypyrone A	*D. gulyae*	*Carthamus lanatus*	[95]
Gulypyrone B	*D. gulyae*	*Carthamus lanatus*	[95]
isofusicoccin	*P. amygdali* (syn. *D. amygdali*)	-	[110]
Kongiidiazadione	*D. kongii*	*Carthamus lanatus*	[101]
monodeacetylfusicoccin	*P. amygdali* (syn. *D. amygdali*)	-	[110]
Nectriapyrone	*D. kongii* *D. eres* *P. foeniculi* (syn. *D. angelicae*)	*Carthamus lanatus* *Vitis vinifera* *Foeniculum vulgare*	[101,105]
Phomentrioloxin B	*D. gulyae*	*Carthamus lanatus*	[95,100]
Phomopsolide B	*Phomopsis* sp. (syn. *Diaporthe* sp.)	*Vitis vinifera*	[66]
Phomopsolidone A	*Phomopsis* sp. (syn. *Diaporthe* sp.)	*Vitis vinifera*	[66]
Phomopsolidone B	*Phomopsis* sp. (syn. *Diaporthe* sp.)	*Vitis vinifera*	[66]
Phomopsin A	*P. leptostromiformis* (syn. *D. toxica*)	*Lupinus* sp.	[113]
Phomozin	*P. helianthi* (syn. *D. helianthi*)	*Helianthus annus*	[114]
α-pyrone convolvulopyrone	*P. convolvulus* (syn. *D. convolvuli*)	*Convolvulus arvensis*	[109]
Sydowinin A	*Phomopsis sp.* (syn. *Diaporthe* sp.)	*Vitis vinifera*	[66]
Sydowinol	*Phomopsis sp.* (syn. *Diaporthe* sp.)	*Vitis vinifera*	[66]
Tyrosol	*D. eres*	*Vitis vinifera* *Hedera helix*	[103,105]

**Table 3:** Benefits of endophytic *Diaporthe* to agriculture.

Host Plant	Endophyte	Benefits	References
*Acampe praemorsa*	*D. eucalyptorum*	Increase the fresh-weight and enhance the growth of ornamental orchids (*Dendrobium* sp.)	[126]
*Combretum lanceolatum*	*D. phaseolorum*	Improve the biosynthesis of primary metabolites involved in *Combretum lanceolatum* self-defence	[127,128]
*Festuca rubra*	*Diaporthe* sp.	Improve nutrient uptake, growth, and salinity tolerance of *Lolium perenne* and the hybrid from *Triticum durum × Hordeum*	[124]
*Helianthus tuberosus*	*D. phaseolorum*	Enhance the growth and increases chlorophyll content of sunchoke plants (*Helianthus tuberosus*)	[11]
*Justicia brandegeana*	*D. masirevicii*	Growth-promoting effects on tomato plants and suppression of *F. oxysporum* symptoms in this host	[129]
*Lactuca sativa*	*P. amygdali* (syn. *D. amygdali*)	Cell enlargement, cotyledon growth and seed germination	[130,131]
*Noccaea goesingensis*	*D. eres*	Improve the biomass *Noccaea goesingensis* and increase nickel accumulation. Potentially used as biofertilizer.	[124]
*Oryza sativa*	*P. liquidambaris* (syn. *D. liquidambaris*)	Enhance growth of rice plants under nitrogen-deficient conditions, and induces rice resistance to rice spikelet rot disease caused by *Fusarium graminearum*	[121]
*Piper nigrum*	*Phomopsis* sp. (syn. *Diaporthe* sp.)	Enhance growth of rice plants due to the production of gibberellins and indole acetic acid	[132]
*Terminalia arjuna* *Phlogacanthus thyrsiflorus*	*D. phaseolorum*	Increase root length and enhance plant growth of *Cicer arietinum*	[133]
*Triticum durum × Hordeum chilense*	*Diaporthe* sp.	Increase concentrations of calcium, magnesium, sulphur, iron, and boron, thus increasing root and shoot biomass of *T. durum × H. chilense*	[122]
